# Effectiveness, feasibility, and acceptability of behaviour change tools used by family doctors: a global systematic review

**DOI:** 10.3399/BJGP.2022.0328

**Published:** 2023-02

**Authors:** Lauren Ball, Bryce Brickley, Lauren T Williams, Jenny Advocat, Elizabeth Rieger, Raeann Ng, Nilakshi Gunatillaka, Alexander M Clark, Elizabeth Sturgiss

**Affiliations:** The University of Queensland, Saint Lucia, Australia.; Menzies Health Institute Queensland, Griffith University, Queensland, Australia.; Menzies Health Institute Queensland, Griffith University, Queensland, Australia.; School of Primary and Allied Health Care, Monash University, Victoria, Australia.; Australian National University, Canberra, Australia.; School of Medicine, Monash University, Victoria, Australia.; School of Primary and Allied Health Care, Monash University, Victoria, Australia.; Faculty of Health Disciplines, Athabasca University, Alberta, Canada.; School of Primary and Allied Health Care, Monash University, Victoria, Australia.

**Keywords:** physicians, family, general practice, lifestyle behaviours, health behaviour, primary care, quality

## Abstract

**Background:**

Priority patients in primary care include people from low-income, rural, or culturally and linguistically diverse communities, and First Nations people.

**Aim:**

To describe the effectiveness, feasibility, and acceptability of behaviour change tools that have been tested by family doctors working with priority patients.

**Design and setting:**

A global systematic review.

**Method:**

Five databases were searched for studies published from 2000 to 2021, of any design, that tested the effectiveness or feasibility of tangible, publicly available behaviour change tools used by family doctors working with priority patients. The methodological quality of each study was appraised using the Mixed Methods Appraisal Tool.

**Results:**

Thirteen of 4931 studies screened met the eligibility criteria, and described 12 tools. The health-related behaviours targeted included smoking, diet and/or physical activity, alcohol and/or drug use, and suicidal ideation. Six tools had an online/web/app-based focus; the remaining six utilised only printed materials and/or in-person training. The effectiveness of the tools was assessed in 11 studies, which used diverse methods, with promising results for enabling behaviour change. The nine studies that assessed feasibility found that the tools were easy to use and enhanced the perceived quality of care.

**Conclusion:**

Many of the identified behaviour change tools were demonstrated to be effective at facilitating change in a target behaviour and/or feasible for use in practice. The tools varied across factors, such as the mode of delivery and the way the tool was intended to influence behaviour. There is clear opportunity to build on existing tools to enable family doctors to assist priority patients towards achieving healthier lifestyles.

## INTRODUCTION

Lifestyle behaviours, such as diet and alcohol intake, physical activity, and smoking, contribute to a substantial proportion of avoidable morbidity and mortality,^1^ so promoting positive behaviour change is a core tenet of family medicine. The United States Preventive Services Task Force reports that, by using behavioural interventions, family doctors (also known as GPs) can support people to meet physical-activity recommendations (32% higher odds than usual care), increase their fruit and vegetable intake by up to 2.2 servings per day, and reduce energy intake by up to 500 kcal per day.^2^^–^5 These positive effects occur in addition to those conferred by medication, can be sustained for at least 12 months, and reduce adverse health events.^5^ Consultation strategies to support doctors and patients in behaviour change are, therefore, important.

Priority patients in primary care include people from low-income, rural, or culturally and linguistically diverse communities, and First Nations people. Providing effective care to these groups is a moral and equitable imperative for family medicine.^6^ Strengthening care for priority patients may maximise potential improvements in population health, given that these groups generally have less opportunity and support than other populations to sustain helpful lifestyle behaviours.^7^ A systematic review and meta-analysis^8^ has shown that behavioural interventions in family medicine settings for priority patients can achieve clinically meaningful improvements in diet (standardised mean difference [SMD] 0.22, 95% confidence interval [CI] = 0.14 to 0.29) and physical activity (SMD 0.21, 95% CI = 0.06 to 0.36), with changes maintained over time. Goal setting and increasing social support can further improve diet and smoking rates for priority patients.^9^

Motivational interviewing with priority patients can reduce alcohol use and substance misuse, while aiding with smoking cessation, physical activity, and sexual health.^10^ Empathy, trust, and an effective doctor–patient relationship can improve the quality of care and support behavioural change and health-related outcomes, including among priority patients.^11^^,^^12^ Clearly, efforts by doctors to support patients who are disadvantaged to improve their lifestyle behaviours are ethically and scientifically justified.

**Table:** How this fits in

Behaviour change is a core tenet of family medicine, and behaviour change tools enhance patients’ intrinsic motivation, confidence, knowledge, and skills to take action and improve their health behaviours. This study systematically reviewed literature to identify behaviour change tools available for use by family doctors when caring for priority patients, including people from low-income, rural, or culturally and linguistically diverse communities, and First Nations people. Twelve heterogenous tools were identified, many with demonstrated effectiveness and feasibility. These tools are an opportunity to support family doctors to help priority patients achieve healthier lifestyles.

The psychological science of behaviour change focuses on the mechanisms and processes that explain how individuals deliberately change their behaviours.^13^ Several health professions, including dietetics, exercise physiology, and social work, incorporate behaviour change science into their practice or professional standards.^14^^–^16 Tools for use in consultations (such as questionnaires, resources, and aides) are common components of high-quality behaviour change practice and are used across behaviour change counselling activities such as motivational interviewing.^17^ Doctors acknowledge the need for greater support to initiate behaviour change conversations in practice, particularly when caring for priority patients;^18^ however, the extent to which behaviour change tools are used by family doctors globally in practice is unknown.

Behaviour change tools are intended to enhance patients’ intrinsic motivation, confidence, knowledge, and skills to take action and improve their health behaviours.^9^ The design, use, and implementation of such tools by family doctors are especially important for priority patients, who experience additional barriers to behaviour change such as a lack of agency.^9^ Given the high promise of behaviour change tools in enhancing quality care and health outcomes, there is a critical need to identify and collate those that have been evaluated for use by doctors working with priority patients. This systematic review aimed to describe the effectiveness, feasibility, and acceptability of the behaviour change tools that have been tested by family doctors working with priority patients.

## METHOD

### Overview

The steps and reporting of this systematic review were conducted in accordance with the Preferred Reporting Items for Systematic Reviews and Meta-Analyses (PRISMA) 2020 statement.^19^ The review followed five steps:

- development of the research question;
- development of the search strategy;
- identification of relevant studies;
- data extraction and article appraisal; and
- data synthesis, and collation and reporting of the results.

The population, intervention, setting, outcomes (PICO) framework^20^ informed the development of the research question: ‘what is known about the effectiveness and feasibility of behaviour change tools used by family doctors caring for disadvantaged patients?’. The systematic review protocol was registered with PROSPERO (reference: CRD42021282175) prior to the literature search.

### Inclusion and exclusion criteria

The PICO framework^20^ was used to develop the criteria from which article eligibility was assessed. To be as inclusive as possible, empirical studies from qualitative, quantitative, and mixed-method research paradigms were included. Non-empirical studies (that is, studies that collected no primary patient data) such as protocol, narrative, and perspective articles were excluded. A publication date range limiter was applied to optimise the availability of identified tools and relevance to the current healthcare system and community context. All inclusion and exclusion criteria are detailed in Table 1.

**Table 1.** Inclusion and exclusion criteria

**Domain**	**Inclusion criteria**	**Exclusion criteria**
**General criteria**	Publication date: January 2000–September 2021 Any scholarly (peer-reviewed) empirical study design — for example, randomised controlled trials, quantitative surveys, and qualitative interviews Human research	Publication date: pre-2000 Non-empirical articles — for example, commentaries and opinion articles
**Population**	Involve a GP/family doctor and a population described as ‘priority’ — for example, indigenous, low-income, and culturally and linguistically diverse	No clear description of clinician involved Generalist physician practising in a hospital setting Involving health professionals but no GP No clear description of priority patient population
**Intervention^a^**	A tangible, publicly available behaviour change tool or product that is not considered part of usual care	Behaviour change theory (for example, social cognitive theory), rather than a tool Behaviour change approach (for example, counselling) rather than a tool Screening tools without any behaviour change components
**Setting**	Primary health care General practice Family medicine Aboriginal medical services	Acute care Hospitals
**Target behaviour**	Health-related behaviours (for example, diet, smoking, and physical activity) and/or family doctor practices	Healthcare screening behaviours (for example, cancer screening) Utilisation of healthcare services Not informing a change in behaviour
**Outcomes**	Must evaluate effectiveness (such as rates of smoking cessation and patient weight loss) and/or feasibility/acceptability, such as perceived usefulness of the tool, perceived relevance, and impact on practice	No evaluation and clear description of tool effectiveness and/or feasibility/acceptability

a *Although the review was not restricted to intervention studies, all included studies were required to report on use of a tool by family doctors.*

### Literature search

Researchers collaborated with a health librarian (blinded for the review) to develop the search strategy, in which initial search term combinations were tested for the retrieval of target articles. The main concepts explored were family doctors, behaviour change tools, and populations experiencing disadvantage (for example, First Nations peoples and groups that are culturally and linguistically diverse).

The following electronic databases were searched in September 2021: MEDLINE (Ovid), Embase, Scopus, APA (American Psychological Association) PsycArticles (Ovid), and CINAHL Complete (EBSCOhost). Medical subject headings were identified for each term and then incorporated into the literature search for the applicable database. Other known synonyms were added to the search, with the Boolean connectors ‘AND’ and ‘OR’ used to combine the search terms.

Proximity searching was implemented to support the search. The articles identified by the search were exported into EndNote, then uploaded to Covidence (with duplicates automatically removed) for screening and data extraction.

### Screening, data extraction, and appraisal

**Figure 1.**
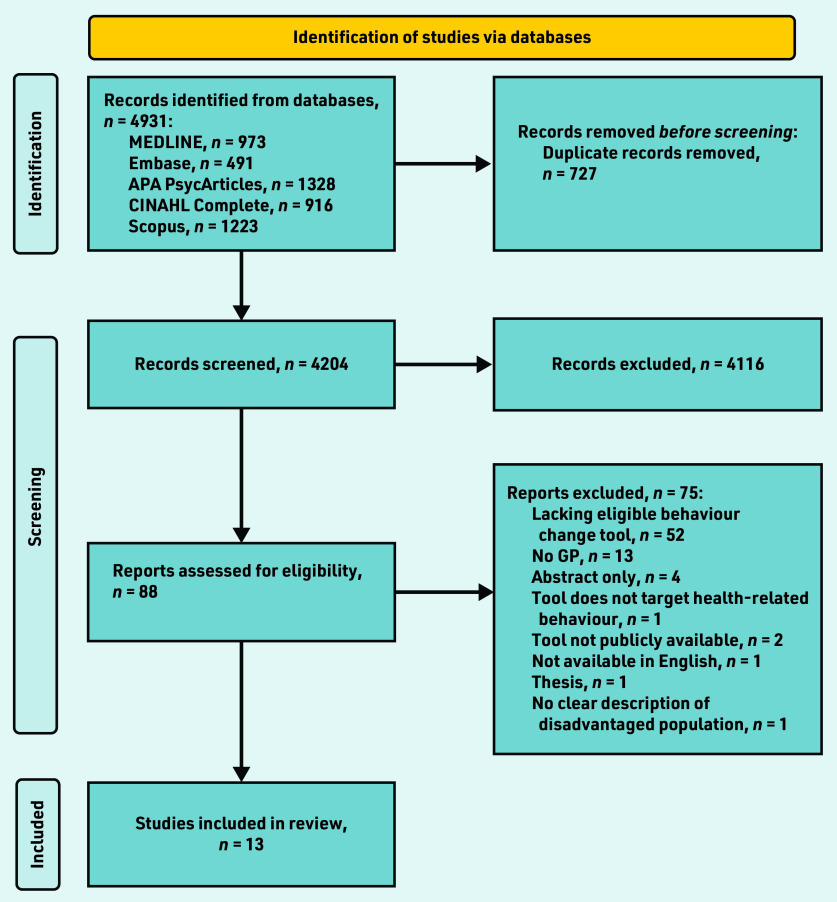
*PRISMA flow diagram. APA = American Psychological Association.*

In Covidence, two researchers independently screened the title and abstract of 50 articles (based on title) for inclusion in accordance with the inclusion criteria, then met with a third researcher to discuss inconsistencies. Next, these three researchers, along with another two, screened the title and abstracts of articles (all retrieved articles, in line with the PRISMA flow chart in Figure 1, *n* = 4204) in accordance with the inclusion criteria. If two researchers agreed on the inclusion of an article, it was screened in full text to determine its eligibility for inclusion. Any conflicts that arose were resolved through reflection and discussion between two researchers. All five of those researchers, along with one other, screened articles in full text; if two researchers agreed on an article’s eligibility it was included for data extraction.

Data from the included articles were extracted by the first two researchers into a common table that was developed *a priori* by the research team. Only data from applicable sections of studies were extracted; if information was inadequate, the corresponding author was contacted for further details.

Data extracted were first author, publication year, country, study aim, study design, description of behaviour change tool, description of population, participants/target clinician in primary care, description of targeted behaviour, effectiveness outcomes, and feasibility outcomes.

The third researcher cross-checked the data extraction by reviewing the table contents in Microsoft Word and each included article. The Mixed Methods Appraisal Tool (MMAT) (version 2018), which allows for the appraisal of studies from various research paradigms, was used to critically appraise articles to assess any risk of bias and indicate certainty or confidence of the evidence reported in individual studies.^19^ The MMAT was used to appraise the included articles’ sampling strategy, appropriateness of methodological approach, representativeness of target population, and trustworthiness of data presentation and interpretation.

### Data analysis

A quantitative meta-analysis was not appropriate for this study, due to the heterogeneity of behavioural variables within the included articles; rather, a meta-synthesis approach^21^^,^^22^ was applied, which comprises:

- identifying the key components of the behaviour change tools being researched;
- providing a narrative account of the contribution (if any) made by each research article, then through added reflection; and
- summarising the overall messages from the literature and discussing them within the context of recommendations of policy, practice, and future research.

## RESULTS

The initial database search identified 4931 articles; a total of 13 studies were identified from the search as being fully eligible for inclusion (Figure 1).^23^^–^35 The main reasons for excluding publications were that the article did not describe an appropriate behaviour change tool or did not involve a family doctor.

One study^36^ was excluded as it had published preparatory work for the tool but had not tested its effectiveness or feasibility. A further two studies^37^^,^^38^ were excluded after writing to the corresponding authors due to the inaccessibility of the behaviour change tool and receiving nothing from the corresponding authors.

A concise summary of the tools identified in the included studies is shown in Table 2.

**Table 2.** Summary of identified tools

**Name of tool**	**Target behaviour(s)**	**Priority population(s)**	**Effectiveness explored?**	**Feasibility explored?**	**Acceptability explored?**	**Patient perspective explored?**
Indigenous Counselling and Nicotine (ICAN) Quit	Smoking	Pregnant Aboriginal and Torres Strait Islander people	✓	✓	✓	✗
Track intervention	Behaviours contributing to obesity	People with multimorbidity and low socioeconomic status	✓	✓	✗	✗
Video Doctor	Eating behaviours and physical activity	Ethnically diverse, low-income pregnant women	✓	✓	✓	✗
Tailored feedback	Health risk behaviours, including smoking, alcohol use, and diet	Non-Aboriginal and Aboriginal people attending an Aboriginal and Community Controlled Health Service	✓	✗	✓	✓
Partnership to Improve Diabetes Education (PRIDE)	Diabetes self-management	People with uncontrolled diabetes attending clinics that serve predominantly uninsured populations with multiple socioeconomic challenges	✓	✗	✓	✗
Childhood Healthy Behaviors Intervention (CHBI)	Eating behaviours, physical activity, and screen time	Children aged 2–9 years with overweight and obesity	✓	✓	✓	✗
Hopple Street Neighborhood Health Center Clinical Innovation Project	Lifestyle behaviours: nutrition and physical activity	Children aged 24–66 months with overweight and obesity	✓	✗	✗	✗
Unnamed tool	Use of alcohol and other drugs use	People with low socioeconomic status	✓	✓	✓	✗
Suicide Prevention Toolkit for Rural Primary Care	Suicidal behaviour	People in rural areas at risk of suicide	✓	✗	✓	✗
Obesity in Children Action Kit	Eating, physical activity, and self-management behaviours	Children aged 2–18 years across targeted paediatric and family physician practices that serve populations with socioeconomic challenges	✓	✓	✓	✗
Partners in Health (PIH) scale; Cue and Response (C&R) Scale	Lifestyle behaviours and self-management of chronic disease	People with chronic disease attending an Aboriginal and Community Controlled Health Service	✗	✓	✗	✗
Tobacco Cessation on Prescription (TCP)	Smoking	Smokers from a region with socioeconomic disadvantage	✗	✓	✓	✓

### Characteristics of included studies

Details of the 13 included studies are given in Supplementary Table S1. Study designs included quantitative randomised controlled trials^23^^–^27 (*n* = 5) and quantitative non-randomised experimental^29^^–^31 (*n* = 3), mixed-methods^28^^,^^32^^,^^33^ (*n* = 3), and qualitative^34^^,^^35^ (*n* = 2) studies. Comparison groups included ‘usual-care’ control groups^23^^–^26^,^^28^^,^^31^^,^^33^ and ‘other intervention’ groups.^26^^,^^27^ Pre/post-studies used within-group baseline measures as a comparator,^23^^,^^30^^,^^33^ and three studies had no comparator.^29^^,^^34^^,^^35^ Most studies were conducted between 2014 and 2020 (*n* = 10),^23^^,^^24^^,^^26^^–^32^,^^35^ and in the US (*n* = 8)^24^^,^^25^^,^^27^^–^30^,^^32^^,^^33^ and Australia (*n* = 3).^23^^,^^26^^,^^34^ The studies ranged in sample size from eight participants^35^ to 520 participants.^28^ The characteristics of priority groups involved in the studies included: First Nations peoples,^23^^,^^26^^,^^34^ migrants from low-income countries, and those with low English-language skills or low health literacy skills;^25^^,^^33^ people with low income and/or socioeconomic status;^24^^,^^25^^,^^27^^–^29^,^^31^ people with multimorbidity;^24^ people from a rural locality;^32^ and young people with chronic disease.^28^^–^30^,^^33^

Patient participants tended to be purposefully selected and recruited into studies based on known characteristics, such as smoking,^23^^,^^35^ pregnancy,^23^^,^^25^ and weight status.^28^^,^^29^ Study durations were relatively short, with only one study including a long-term (24-month) follow-up.^28^

### Description of tools aimed at changing behaviours

Twelve tools were tested in the studies; these are summarised in Table 2, with more detail given in Supplementary Table S2. The health-related behaviours that were targeted were smoking,^23^^,^^25^ diet and/or physical activity,^24^^–^26^,^^28^^,^^29^^,^^33^ alcohol and/or drug use,^31^ and suicidal ideation.^32^ The behaviours were targeted singularly (for example, smoking),^35^ or as a collective (for example, behaviours contributing to obesity).^24^^,^^28^^–^30^,^^33^ Six tools had an online/web/app-based focus,^23^^–^25^,^^31^^–^33 and seven tools (cited in eight studies^26^^–^30^,^^34^^,^^35^^,^^39^) utilised only printed materials and/or in-person training.

Many of the tools aimed to guide clinicians through a series of steps when interacting with patients during consultations; in addition, many were designed for a particular population, such as a specific cultural group, and used intentional, tailored language and pictorials.^25^^,^^27^^,^^33^ Two studies^24^^,^^30^ used elements of follow-up or tracked engagement with patients following a consultation. Only two tools were explicitly informed by a stated behaviour change framework, which were social cognitive theory^24^ and the theoretical domains framework.^23^

The most common behaviour change strategies that were used were goals and planning, feedback and monitoring, social support, shaping knowledge, and repetition and substitution.^40^

### Effectiveness, feasibility, and acceptability

Eleven studies^23^^–^33 assessed the effectiveness of the tool at improving the quality of care provided to patients and/or facilitating behaviour change among patients. Nine studies assessed behaviour change, such as improvements in goal setting,^25^^,^^28^^–^30^,^^33^ increased rates of discussing a specified behaviour with the doctor,^26^^,^^38^ and self-care for mental or physical health and self-efficacy for chronic disease management.^27^^,^^32^ Two studies assessed effectiveness through changes in health characteristics, such as dietary modifications,^25^ weight management, and exercise behaviour.^33^

Evidence of improvements in health behaviours was observed, such as an average increased weekly exercise duration of ∼28 min per week by participants who used the Video Doctor Tool.^25^ Modest evidence of improvements in health outcomes were observed in some studies, such as a statistically significant greater average weight loss among patients who used the Track Intervention for 12 months, compared with those in the usual-care group (−4.0 kg versus −0.1 kg, *P*<0.001).^24^

Nine studies^23^^–^25^,^^28^^,^^29^^,^^31^^,^^33^^–^35 assessed the feasibility of the tool by assessing outcomes, such as the extent to which doctors found the tool easy to use, whether the tool was delivered as intended, whether doctors adopted the tools in practice, and whether the use of the tool was sustained. One study^26^ reported that doctor participants felt the tool was relevant to their clinical practice. Two studies^29^^,^^32^ evaluated feasibility and acceptability from the doctor perspective of whether the tools were likely to influence consultation behaviours, such as doctor–patient communication and behavioural assessment.

Overall, researchers reported their tools to be feasible given that many tools had high implementation rates and were positively perceived by doctors,^24^^,^^25^^,^^33^^,^^37^^,^^39^ indicating that they were successfully utilised in clinical practice. No studies described plans for refining or implementing the tool beyond the study.

### Quality of studies

The methodological quality of the studies was mixed (Table 3); overall, they were assessed to have considerable risk of bias, primarily due to insufficient or inadequate reporting, or being unable to blind study participants, clinicians, and assessors. Only one study^35^ met all possible appraisal criteria; the reporting of this rigorous qualitative study demonstrated coherence between data sources, analysis, and interpretation.

**Table 3.** Article appraisal

**Study**	**Mixed Methods Appraisal Tool criteria**				
**Quantitative randomised controlled trials**					
	2.1	2.2	2.3	2.4	2.5
Bar-Zeev *et al*^23^	Yes	Unknown	No	Unknown	No
Bennett *et al*^24^	Yes	Yes	Yes	No	Yes
Jackson *et al*^25^	Yes	Yes	Yes	N/A^a^	Yes
Noble *et al*^26^	Yes	Yes	No	N/A^a^	Yes
White *et al*^27^	Yes	Yes	No	No	Yes

**Quantitative non-randomised experimental studies**					
	3.1	3.2	3.3	3.4	3.5
Camp *et al*^29^	Unknown	Yes	Yes	Yes	Yes
Herbst *et al*^30^	Yes	Yes	Yes	Yes	Unknown
Salvalaggio *et al*^31^	Yes	Yes	No	Yes	Yes

**Mixed-methods studies**					
	5.1	5.2	5.3	5.4	5.5
Camp *et al* 28	Unknown	Yes	No	No	Yes
McFaul *et al*^32^	Unknown	Yes	Yes	Unknown	No
Sealy *et al*^33^	Unknown	Yes	Yes	Unknown	Yes

**Qualitative studies**					
	1.1	1.2	1.3	1.4	1.5
Abbott *et al*^34^	Unknown	Unknown	Unknown	No	No
Leppänen *et al*^35^	Yes	Yes	Yes	Yes	Yes

a *Patient participants were the outcome assessors as per the study design so it was not possible to blind them to the intervention.*

## DISCUSSION

### Summary

Only 12 tools (from 13 studies) met the eligibility criteria, nearly all of which were published in the last decade; they were used with a wide range of priority patient populations, such as First Nations peoples, people with low socioeconomic status, people without insurance, those living in rural areas, and children experiencing chronic disease.

The identified tools were effective at addressing common barriers family doctors may expect to encounter when delivering behavioural interventions, such as short consultation duration, uncertain capability at facilitating behaviour change with patients, and lack of confidence to raise the topic opportunistically.^18^

Many were also demonstrated to be effective at facilitating change in a target behaviour or were feasible for use in practice by doctors. However, as elaborated below, there was considerable diversity across studies across several factors, including the targeted patient population, mode of delivery, and the way the tool was intended to influence behaviour, as well as notable gaps in the utilisation of behaviour change techniques. Further, the tools appeared to be evaluated by the authors or members of the intervention team, introducing further potential bias to the interpretation of those results. Patient perspectives of the tools were rarely explored in the identified studies.

The studies in this review had several inherent limitations. None reviewed explored cost-effectiveness in enhancing healthcare outcomes, which is an important concept as healthcare services caring for priority population groups may have scarce resources. In addition, the strength and accuracy of the evaluation of each tool is dependent on the characteristics of the design of each included study; however, fewer than half of the identified studies were randomised controlled trials and, of these, only two studies had a true comparator group.

There was considerable variability across studies, which made it difficult to make comparisons; this extended to the tools utilised and the mode in which they were delivered. Tool modality, for example, included online interactive webinar training for doctors, mobile telephone application for patient self-monitoring, and printed tools for the patient and/or doctor. The measures used to assess effectiveness, feasibility, and acceptability also varied across health-related and patient- and clinician-reported outcomes. Despite the variety of tools identified, in each study a family doctor was fundamental to the success of the tool’s use and achieving change in the target behaviour.

### Strengths and limitations

To the authors’ knowledge, this is the first review to identify and synthesise behaviour change tools that have been implemented and evaluated, and are ready for use by doctors when caring for priority patient populations. It provides a comprehensive guide to the tools currently available in general practice. Other strengths of the review include the comprehensive search and selection process, the 20-year timeframe, adherence to quality-assurance standards, and the fact that it was conducted by a multidisciplinary team consisting of behaviour change experts, family doctors, academics, and dietitians. A collaborative approach reduced the introduction of clinical, experience-related biases and resulted in a comprehensive literature synthesis and reporting.

Overall, the review highlighted that there is a clear opportunity to build on existing behaviour change tools to enable change among priority patient populations towards healthier lifestyles. However, organisations that work to support the education and continuing professional development of family doctors may not publish their tools and resources in scholarly literature and, as such, would not have been included in the search.

Although the search criteria limited the tools to those used among priority patient populations, which may have led to the exclusion of tools evaluated in other groups from this review, it serves to demonstrate how few behaviour change tools have been developed and evaluated specifically for priority populations.

### Comparison with existing literature

Unknown efficacy of the tools and limited evaluation were characteristics of studies in a previous literature review of behaviour change tools.^41^ There was also minimal evidence of the use of theoretical models outlining the processes or determinants of behaviour change to guide tool development; this is consistent with a recent scoping review of nutrition interventions, which found that >70% of the interventions in the review failed to report a behaviour change theory.^42^

### Implications for research and practice

Providing care to priority populations requires special consideration to the likely inequities they have experienced in the past — for example, First Nations peoples’ care must be culturally safe.^42^ Clinicians in the samples of this literature synthesis sought to influence patient behaviour by shaping knowledge and, in doing so, listened to patients, fostered relationships, and delivered tailored educational messages, which are concepts consistent with a broad understanding of person-centred care.^43^ The promising effectiveness and feasibility outcomes of the included tools convey the value of a person-centred approach to behaviour change.

There is scope for significant improvement in this body of literature. Exclusion of the patient perspective in the evaluation of behaviour change tools can compromise whether the tools can be readily adopted in primary care. Clearly, more rigorous and independent evaluation of behaviour change tools needs to be conducted over a longer term and in ongoing usual care. Given the promising application of these tools, future research should explore whether there are any barriers and facilitators to the adoption and implementation of behaviour change tools in primary care.

Priority populations have distinct care needs, which can limit the effectiveness of traditional behaviour change interventions (for example, telephone counselling), so the development of tools dedicated for use among these populations is essential.^44^ The tools utilised in the included studies primarily focused on goals and planning, feedback and monitoring, social support, and shaping knowledge in line with guidance on behaviour change taxonomy.^40^ Utilising a limited range of tools may fail to fully capture the complexity of patient-centred general practice care and the processes entailed in facilitating health behaviour change;^45^ as such, future research should explore the development of tools that include a broader range of behaviour change techniques and evaluate their impact when delivered to priority populations.

The use of behaviour change tools that lack adequate theoretical scaffolding may result in ineffective behaviour change attempts, or even have unintended, adverse effects. The tailored-feedback tool that was identified in this review had minimal theoretical foundations; use of it, in practice, can give patients feedback and shape their knowledge — for example, use of it can outline to the patient the health risks associated with a behaviour — but, in the absence of considered follow-up by a GP and any strategies/resources to support behaviour change, it might increase patients’ sense of low self-efficacy and, therefore, work against them taking action to make a change.^44^ Comprehensive theoretical scaffolding within behaviour change tools is, therefore, essential to supporting positive behavioural outcomes.

To support doctors in practice, there is a need to develop a hub that brings together behaviour change tools to encourage their usage among doctors — future work exploring the development of a clinician-facing platform that stores professional development resources and tools that can assist doctors to facilitate behaviour change would be beneficial.

The identified lack of rigorously evaluated and accessible behaviour change tools presents an opportunity to further support family doctors in facilitating behaviour change with priority patients. Although there was much variability across studies, engagement between the doctor, patient, and behaviour change tool demonstrated utility to enable behaviour change discussions. Further research with independent evaluations working alongside psychologists who are experts in behaviour change science is warranted.

A key step towards helping doctors to navigate the complexity of behaviour change would be to synthesise and make explicit the tools supporting behaviour change strategies that are effective in practice and are relevant to them. Accordingly, future work needs to develop an evidence-based, resource-rich hub that can be accessed by doctors when seeking assistance to facilitate behaviour change in priority patients.
